# High affinity binding of the peptide agonist TIP-39 to the parathyroid hormone 2 (PTH_2_) receptor requires the hydroxyl group of Tyr-318 on transmembrane helix 5

**DOI:** 10.1016/j.bcp.2016.12.013

**Published:** 2017-03-01

**Authors:** Richard E. Weaver, Juan C. Mobarec, Mark J. Wigglesworth, Christopher A. Reynolds, Dan Donnelly

**Affiliations:** a School of Biomedical Sciences, Faculty of Biological Sciences, University of Leeds, Leeds LS2 9JT, UK; b GlaxoSmithKline, New Frontiers Science Park North, Third Avenue, Harlow CM19 5AW, UK; c School of Biological Sciences, University of Essex, Wivenhoe Park, Colchester CO4 3SQ, UK

**Keywords:** Parathyroid hormone, PTH, GPCR, Agonist, Receptor, TIP39

## Abstract

TIP39 (“tuberoinfundibular peptide of 39 residues”) acts via the parathyroid hormone 2 receptor, PTH_2_, a Family B G protein-coupled receptor (GPCR). Despite the importance of GPCRs in human physiology and pharmacotherapy, little is known about the molecular details of the TIP39-PTH_2_ interaction. To address this, we utilised the different pharmacological profiles of TIP39 and PTH(1–34) at PTH_2_ and its related receptor PTH_1_: TIP39 being an agonist at the former but an antagonist at the latter, while PTH(1–34) activates both. A total of 23 site-directed mutations of PTH_2_, in which residues were substituted to the equivalent in PTH_1,_ were made and pharmacologically screened for agonist activity. Follow-up mutations were analysed by radioligand binding and cAMP assays. A model of the TIP39-PTH_2_ complex was built and analysed using molecular dynamics. Only Tyr318-Ile displayed reduced TIP39 potency, despite having increased PTH(1–34) potency, and further mutagenesis and analysis at this site demonstrated that this was due to reduced TIP39 affinity at Tyr318-Ile (pIC_50_ = 6.01 ± 0.03) compared with wild type (pIC_50_ = 7.81 ± 0.03). The hydroxyl group of the Tyr-318′s side chain was shown to be important for TIP39 binding, with the Tyr318-Phe mutant displaying 13-fold lower affinity and 35-fold lower potency compared with wild type. TIP39 truncated by up to 5 residues at the N-terminus was still sensitive to the mutations at Tyr-318, suggesting that it interacts with a region within TIP39(6–39). Molecular modelling and molecular dynamics simulations suggest that the selectivity is based on an interaction between the Tyr-318 hydroxyl group with the carboxylate side chain of Asp-7 of the peptide.

## Graphical abstract

**Figure:**
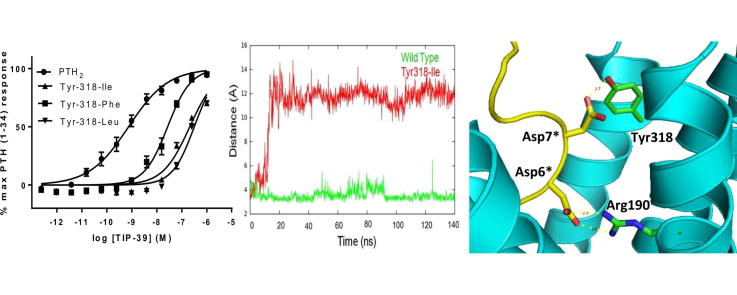

## 1 Introduction

The recent increase in structural information for class B GPCRs, encompassing both the extracellular domain and the transmembrane helical bundle, can be used to interpret pharmacological studies of class B peptide hormones. Here our focus is on the parathyroid hormone 2 receptor (PTH_2_), a Family B G protein-coupled receptor (GPCR) which is potently activated by its endogenous neuropeptide TIP39 (“tuberoinfundibular peptide of 39 residues”). Human PTH_2_ is also activated by parathyroid hormone (PTH) and indeed shares 50% sequence identity with PTH_1_, the receptor for both PTH and PTH-related peptide (PTHrP), which is why PTH_2_ was named after PTH. However, TIP39, acting through PTH_2_, has very distinct physiological roles compared with the calcium homeostasis function of PTH acting through PTH_1_ – for example, TIP39 modulates various aspects of the stress response, and is also involved in thermoregulation, nociception, and prolactin release [1]. Here we seek to identify the key interactions that govern the selective activation of PTH_2_ by TIP39.

Like other Family B GPCRs, PTH_2_ is activated by peptide agonists via a two site interaction model [2], [3] in which the ligand’s C-terminal α-helical region interacts with the receptor’s N-terminal extracellular (N) domain to generate affinity, while the N-terminal region of the peptide activates the receptor via a second interaction with the receptor’s transmembrane helices and connecting loops (juxta-membrane “J” domain). The nature of the first interaction has been detailed via the solution of the structure of the ligand-bound extracellular domain of PTH_1_ via X-ray crystallography [4]. The crystal structure showed that the ligand forms an α-helix which docks into a long hydrophobic groove on the N domain via hydrophobic interactions formed by Val-21^∗^, Trp-23^∗^, Leu-24^∗^, Leu-28^∗^, Val-31^∗^ and Phe-34^∗^ of PTH (ligand residues will be distinguished from receptor residues by an asterisk following the residue number). However, despite the solution of the crystal structure of the isolated J domain of two related family B GPCRs [5], [6], [7], the molecular details of the second activating interaction remain to be determined due to the absence of endogenous ligands in these structures. In the absence of a crystal structure of a peptide-bound Family B GPCR, some insights into how peptides bind to the J domain have nevertheless been gained through protein chemical and molecular pharmacological approaches. For example, the extreme N-terminal residues of both PTH and PTHrP have each been replaced by benzoylphenylalanine (BPA), and these active peptide agonist analogues have been cross-linked to Met-425 of the receptor [8], [9], with a model generated which suggested that the N-terminus of PTH lies across the extracellular surface of the receptor [8]. The cross-linking results were later refined by use of disulphide trapping, which can be more specific than BPA-based photoaffinity cross-linking, implying a preference for contacts between the extreme N-terminus of PTH with Leu-368, Try-421, Phe-424 and Met-425 [10]. The general consensus at that time, which predated the X-ray structures of the TM domain, was that class B receptors may bind peptides in a variety of ways [11], [12], and the peptide binding model based upon the cysteine trapping data was only refined slightly from that derived from the earlier BPA data, with the N-terminus of the peptide interacting with the extracellular face of the TM domain, rather than binding deeper into the core of the helical bundle.

**Fig. 1:**
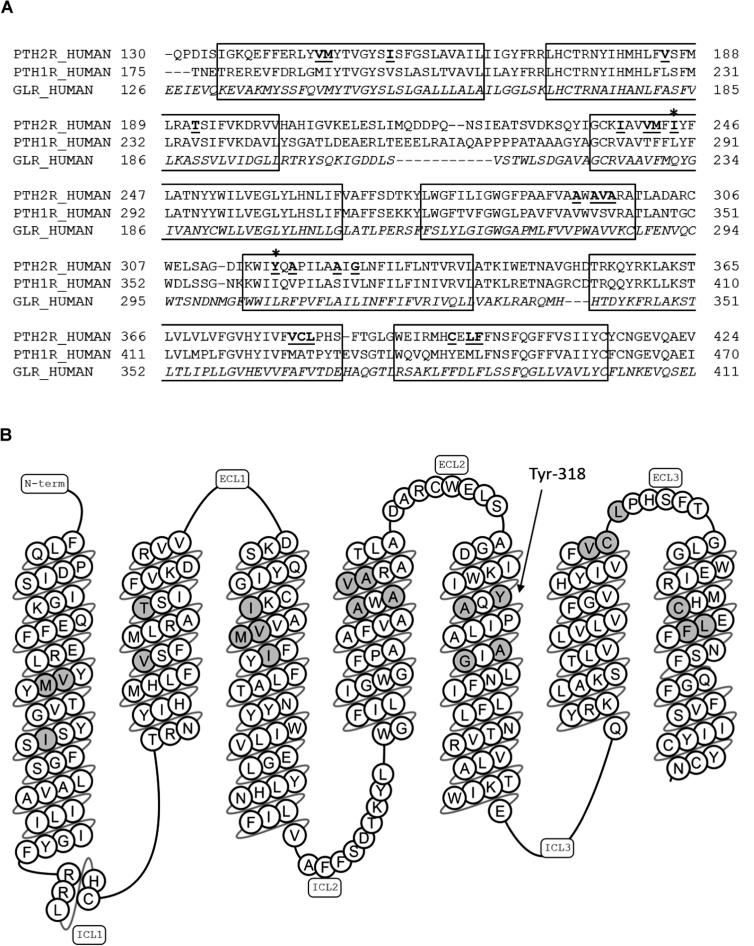
A. Sequence alignment of three Family B GPCRs: PTH_2_ (PTH2R_HUMAN); PTH_1_ (PTH1_HUMAN); and the glucagon receptor (GLR_HUMAN).The seven transmembrane helices are boxed and are based on the crystal structure of the glucagon receptor [6]. The first and last residue number of each sequence is shown at the start and end of each line respectively. The residues in PTH_2_ which were mutated to those of PTH_1_ are shown bold and underlined. B. A schematic topological representation (generated using GPCRDB, http://gpcrdb.org) of PTH_2_, annotated to show the regions mutated in this study (grey) with the residue numbers of the most interesting site highlighted. TM = transmembrane helix; ECL = extracellular loop.

While PTH is able to potently activate both human PTH_1_ and PTH_2_ receptors, surprisingly PTHrP does not activate PTH_2_, despite binding with moderate affinity. By using chimeric receptors and modified peptide ligands, it has been shown that the features responsible for the ability of PTH_2_ to select against PTHrP are due to Ile-5^∗^ and Trp-23^∗^ of PTH being His-5^∗^ and Phe-23^∗^ in PTHrP [13]. The high affinity of PTH at PTH_2_ is maintained in part by the interaction between Trp-23^∗^ of the peptide and Val-41 in the N-domain of the receptor [4], [14]. However, this interaction is absent for PTHrP/PTH_2_ binding, due to the smaller size of Phe-23^∗^ [14], which results in lower affinity. The inability of PTHrP to activate PTH_2_ is due to the presence of His-5^∗^ in PTHrP, rather than Ile-5^∗^ in PTH, which has been functionally linked through two reciprocal receptor studies to Ile-244 and Tyr-318 in the J domain of PTH_2_ [13], [15]. Interestingly, the two topologically equivalent residues to Ile-244 and Tyr-318 in the glucagon receptor (Gln-232 and Leu-307) can be found to be in close contact with each other in the crystal structures of the latter [6], [7]; see Fig. 1A for a sequence alignment). In contrast to what has been previously suggested for PTH [10], the location of Ile-244 and Tyr-318 within the TM bundle implies that the N-terminus of PTH receptor ligands may bind within the TM domain. This would be in line with what has been suggested for peptide binding at the glucagon and GLP-1 receptors through detailed and extensive mutagenesis and modelling studies [6], [16], [17], [18], [19].

Despite the success of the chimeric and single residue-swap studies of PTH_1_ and PTH_2_ described above [13], [14], [15], [20], which identified PTH/PTHrP binding and selectivity determinants at PTH_1_ and PTH_2_, the nature of the TIP39 selection has not been explored to the same degree. While TIP39 is a potent agonist at PTH_2_, it does not activate PTH_1_, despite binding with moderate affinity [21]. Chimeric PTH_1_/PTH_2_ receptors have been used to demonstrate that the J domain of PTH_1_ is responsible for selecting against the high affinity binding of TIP39 and that this domain is likely to interact with the N-terminal region of the ligand [21]. The truncation of the first 6 residues of TIP39, to yield TIP (7–39), resulted in a peptide with no efficacy at PTH_2_ but increased its affinity at PTH_1_ relative to TIP39 [21], suggesting that selectivity for PTH_2_ activation is encoded within the first 6 residues of peptide.

The aim of this study was to use site-directed mutagenesis to substitute selected PTH_2_ residues in the J domain, with those found in PTH_1_, in order to identify residues in PTH_2_ that play a role in ligand selection through the recognition of the N-terminal region of TIP39. To aid the interpretation of the data generated from the study, and to resolve the argument as to whether the PTH receptor ligands bind more deeply within the TM domain, we constructed a 3-dimensional model of PTH_2_ receptor bound with TIP39, based upon previous models of agonist-bound GLP-1R, and analysed the residues that could interact with Tyr-318 using all-atom molecular dynamics simulations.

## 2 Methods

### 2.1 Constructs

The full-length cDNA of human PTH_1_ and PTH_2_ (gift from GlaxoSmithKline) in pcDNA3 (Invitrogen, Paisley, UK) were used to express wild type receptors as described previously [14]. Mutant PTH_2_ receptors were selected (Fig. 1) and generated using QuikChange® site-directed mutagenesis (Stratagene, La Jolla, CA, USA) and confirmed by DNA sequencing. These various pcDNA3 constructs were used to express the wild type human PTH_1_ and PTH_2_ receptors, and mutant PTH_2_ receptors, in Human Embryonic Kidney (HEK)-293 cells. Residues which were predicted to be close to the extracellular ends of the TM regions of PTH_2_, and which were not conserved between PTH_1_ and PTH_2_, were targeted for site-directed mutagenesis.

### 2.2 Cell culture

The HEK-293 cells were cultured in Dulbecco’s Modified Eagle’s Medium (DMEM, from Sigma, Poole, UK) supplemented with 10% foetal calf serum (Lonza Wokinham Ltd., Wokingham, UK) and containing 2 mM L-glutamine, 100 U/ml penicillin and 100 μg/ml streptomycin (Invitrogen, Paisley, UK). Cells were transfected with pcDNA3 containing the cDNA encoding the receptors, using the SuperFect® Transfection Reagent (Qiagen Ltd., Crawley, UK.) and stable clones were selected with G418 antibiotic (Invitrogen, Paisley, UK) as follows. Cells were seeded into a 25 cm^2^ flask containing 10 ml of media and transfected when they reached 50–80% confluence. To do this, 20 μl of SuperFect® was mixed with a DNA solution consisting of 5 μg plasmid DNA in 150 μl DMEM. The DNA was incubated with the reagent for 10 min at room temperature after which 1 ml of media was then added and mixed gently. The cells were washed once with sterile PBS (137 mM NaCl, 10 mM Phosphate, 2.7 mM KCl, pH 7.4; Sigma, Poole, UK) before the transfection mixture was added and incubated for 3 h at 37 °C. The cells were then washed 3 times with PBS before the addition of fresh media. Three days later, the supernatant was removed and the cells were washed with PBS before fresh media was added. Selection of transfected cells was achieved by addition of 800 μg ml^−1^ G418. The media, containing G418, was replaced every 3 days until individual colonies were clearly visible. Approximately 10–20 individual colonies were detached from the flask using trypsin, seeded in a fresh plate and grown to confluence.

### 2.3 Peptides

PTH(1–34), TIP39 and rat [Nle^8,21^,Tyr^34^]rPTH(1–34)NH_2_ [called rPTH(1–34) throughout this paper] were from Bachem (Saffron Walden, UK). [Trp^23^,Tyr^36^]PTH(1–36) was custom synthesised by Cambridge Research Biochemicals. The five truncated TIP39 peptides (TIP (2–39), TIP (3–39), TIP (4–39), TIP (5–39), all with free carboxyl C-termini, were custom synthesised by Genosphere Biotechnologies (Paris, France) to >95% purity as analysed via RP-HPLC (at 220 nm) and Mass Spec. The radioligand ^125^I-[Nle^8,21^,Tyr^34^] rat PTH(1–34)NH_2_ (called ^125^I-rPTH(1–34) throughout the paper) was from Perkin Elmer Life and Analytical Sciences (Waltham, MA, USA).

### 2.4 Radioligand binding

HEK-293 cells expressing the receptor(s) of interest were grown to confluence in poly-D-lysine coated 24-well plates. Radioligand and unlabelled peptides were made up in ‘Whole Cell Binding Buffer’ (WCBB: 100 nM NaCl, 50 mM Tric-HCl, 5 mM KCl, 2 mM CaCl_2_ pH 7.7; Sigma, Poole, UK) supplemented with 5% heat inactivated horse serum and 0.5% heat inactivated foetal calf serum. The cell culture media was removed and 150 μL of ^125^I-rPTH(1–34) was then added to each well to give a final concentration of 50 pM (∼50,000 cpm). 150 μL of serially diluted unlabelled ligand (10 μM–10 pM) were then added to each well and the cells incubated at room temperature for 2 h. The cells were then washed three times with WCBB, lysed with 500 μL of 5 M NaOH, and then the radioactivity in the cell lysate was measured in a gamma counter.

### 2.5 cAMP accumulation assay

The LANCE cAMP kit (PerkinElmer Life and Analytical Sciences) was used alongside the manufacturer’s instructions with some minor adaptations as described. Cells were washed and resuspended in Stimulation buffer (HBSS, 5 mM HEPES, 0.1% BSA, 500 μM IBMX, pH 7.4; Sigma, Poole, UK) to the required concentration. The ligands were prepared in DMSO at 100-fold stimulation concentrations and 0.1 μL added to each well of a white 384-well low volume OptiPlate. 5 μL of cells were added to each well followed by 5 μL of Stimulation buffer containing the Alexa Fluor® 647-labelled antibody, and incubated at room temperature. After the cell stimulation period, 10 μL of Detection mix was added to each well and incubated for 1 h at room temperature. The data shown in Table 1, Table 2, Table 3 were generated by REW either at GSK (Table 1, Table 2) or Leeds (Table 3). The preliminary screening data in Table 1 were derived using assay conditions of 10,000 cells/well and a ligand stimulation time of 30 min, with 0.005 (v/v) Alexa Fluor®. The acceptor fluorescence signal was read at 665 nm on a ViewLux instrument (Perkin Elmer). Based on the results of this screen, further mutagenesis of Tyr-318, followed by stable cell line generation, were carried out and the full dose–response data shown in Table 2 were generated using 5000 cells/well and a ligand stimulation time of 20 min – the change in conditions was required to reduce the assay sensitivity in order to fit within the window of the standard cAMP curve generated using the assay kit at that time. The truncated TIP39 data shown in Table 3 were generated at a later stage in Leeds and the conditions were also modified in order to fit within the window of the standard cAMP curve: 2500 cells/well and a ligand stimulation time of 10 min, with 0.0025 (v/v) Alexa Fluor®, read at 665 nm (Victor X4 plate-reader, Perkin Elmer). Note that data have only been directly compared *within* each table for which assay conditions were identical.

### 2.6 Data analysis

For each individual competition binding experiment, counts were normalised to the maximal specific binding within each data set. IC_50_ values were calculated with a single site binding model with the Hill co-efficient constrained to 1, while EC_50_ values were calculated with a symmetrical sigmoid function, using non-linear regression with the aid of PRISM 5.0 software (GraphPad Software San Diego, CA, USA). Values in the tables represent the mean with S.E.M. of the individual pIC_50_ (Log IC_50_) or pEC_50_ values from at least three independent experiments, each of which was carried out with triplicate vales for each ligand concentration. Comparisons with controls were assessed using a two-tailed unpaired *t* test using GraphPad. Curves in the figures represent pooled data from three independent experiments where each point is the mean of the normalised values with the inter-experimental standard error of the mean displayed as error bars. B_max_ values were calculated from rPTH(1–34) homologous binding assays using B_max_ = B_0_ × IC_50_/[L], where [L] is the concentration of free radioligand and B_0_ is the specific binding in the absence of unlabelled ligand. B_max_ values were expressed as fmol of receptor per mg of membrane protein where the latter was calculated using a bicinchoninic acid protein assay using bovine serum albumin to create a standard curve.

### 2.7 Modelling method

#### 2.7.1 Agonist-bound PTH_2_ receptor model

All molecular modelling manipulations were carried out using the tools embedded within PyMOL (The PyMOL Molecular Graphics System, Version 1.7.2.3 Schrödinger, LLC) unless otherwise stated. The first stage was to make a homology model of the J domain of PTH_2_ from the crystal structure of the J domain of the glucagon receptor ([6]; pdb code 4L6R) using the homology modelling server SWISS-MODEL ([22]; http://swissmodel.expasy.org/). In an independent step, the ligand within the PTH-bound N domain crystal structure of PTH_1_ ([4]; pdb code 3C4M) was mutated *in silico* to the sequence of TIP39 (starting at Ala-15^∗^) and the N domain of PTH_2_ was built from 3C4M using SWISS-MODEL. Since the ligand co-ordinates were stripped out during the homology modelling stage, these TIP39 and PTH_2_ fragments were then re-docked by superposing them back on the 3C4M template. The Dods and Donnelly model [16] of the agonist-bound GLP-1 receptor was then used as a scaffold from which the full-length TIP39-bound PTH_2_ was constructed by first superimposing the PTH_2_ J domain model onto the corresponding 7TM region of GLP-1R, and then superimposing the TIP39 ligand on the GLP-1 peptide and, in doing so, orientating the N domain relative to the J domain. The merged fragments were saved as a single pdb file and then used as a template in SWISS-MODEL from which PTH_2_ was rebuilt, enabling the linker region between the domains to be constructed as a loop. Since the ligand co-ordinates were stripped out during the homology modelling stage, TIP39 was re-docked according to the 3C4M template and then manually extended along the GLP-1R peptide model trajectory. Two main constraints were used to guide the latter stage: (1) since Arg-190 and Lys-197 are conserved between GLP-1R and PTH_2_, and are believed to interact with Glu-3 in the former [16], [17], [18], [19], the equivalent residue in TIP39 (Asp-6) was positioned in a similar position; (2) Ala-3 (equivalent to the first residue of PTH) was placed close to residues 376, 379 and 380, since the equivalent residues in PTH_1_ have been cross-linked to the extreme N-terminus of PTH [10]. The first loop of the N domain was constructed using PLOP [23] and model was then subjected to optimisation using the KoBa^MIN^ server ([24]; http://csb.stanford.edu/kobamin) to yield the starting model for molecular dynamics simulations.

#### 2.7.2 Molecular dynamics simulations

Simulations of PTH_2_ in complex with two variants of TIP39 were prepared as follows. All the PTH_2_-TIP-39 complexes were embedded in a POPC bilayer with explicit water and ions to a final concentration of 150 mM, and simulated using ACEMD [25], with the AMBER 14SB [26] and lipid 14 force fields [27]. The simulation protocol included standard steps of energy minimization, heating from 0 to 300 K, and progressive decrease of conformational constraints followed by the unconstrained production run. Simulation 1 contained the full TIP39 peptide and the production run was 442 ns. However, due to uncertainties in predicting the starting conformation of the N-terminal region of full length TIP-39, we decided to simulate variants of TIP39 with a truncated N-terminus. Simulation 2 contained PTH_2_ bound to TIP (5–39), and the production run was 250 ns. Simulation 3 contained PTH_2_ with the Tyr318-Ile mutation and TIP (5–39) peptide, the production run was 140 ns. The initial systems dimensions and sizes were 88.31 Å × 88.68 Å × 138.24 Å with 98,594 atoms for simulation 1, 88.03 Å × 88.39 Å × 134.24 Å with 95,089 atoms for simulation 2, and 88.31 Å × 88.39 Å × 142.24 Å with 101,659 atoms for simulation 3. Hydrogen bonds were defined with a donor–acceptor distance <3.0 Å, and an angle cut-off of 20°. The models and trajectories are available from ftp.essex.ac.uk/pub/oyster.

## 3 Results

### 3.1 Initial pharmacological screen

A total of 25 stable cell lines were created for the initial screen, expressing either wild type PTH_1_, PTH_2_, or one of 23 mutant PTH_2_ receptors. Table 1 shows the response generated from LANCE cAMP assays using concentrations of PTH(1–34) and TIP39 which had been shown to generate maximal responses in full concentration response experiments using these particular assay conditions (data not shown). As expected, PTH(1–34) was able to fully activate both PTH_1_ and PTH_2_, as well as all the 23 single mutant PTH_2_ receptors. TIP39 was unable to activate PTH_1_ but displayed maximal activity at 22 of the 23 mutant PTH_2_ receptors, the exception being Tyr-318-Ile which displayed only about 50% maximal activity.

### 3.2 Tyr-318-Ile

**Fig. 2:**
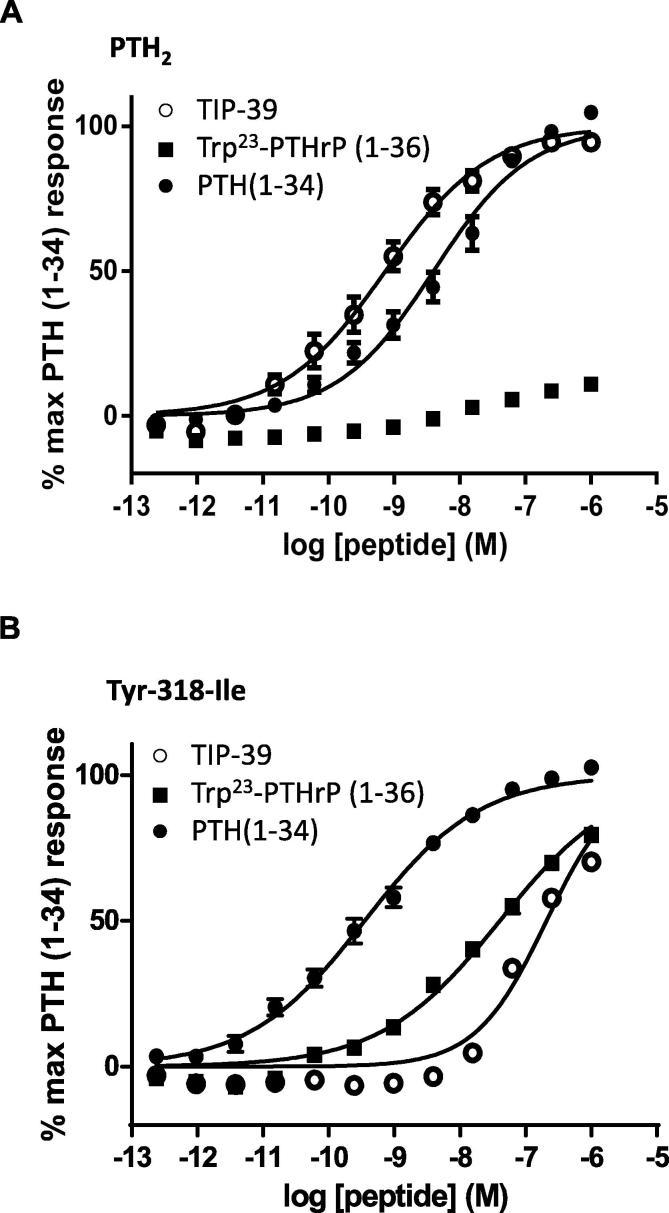
cAMP accumulation curves for HEK-293 cells expressing A PTH_2_ and B Tyr-318-Ile, using three peptide ligands as indicated in the key. Curves represent pooled data from three independent experiments where each point is the mean of the normalised values and inter-experimental standard error of the mean is displayed as error bars.

The mutant PTH_2_ receptor, Tyr-318-Ile, was examined in more detail by using whole-cell radioligand binding assays and by generating full concentration–response curves using PTH(1–34), TIP39 and the affinity-optimised PTHrP analogue, Trp23-PTHrP (Table 2 and Fig. 2). The typical pharmacological profile of PTH_2_ was observed (Fig.2A), with high potency displayed for PTH(1–34) and TIP39 but no activity with Trp23-PTHrP. However, the pharmacological profile for Tyr-318-Ile was radically different (Fig.2B). As shown previously [15], [20], Tyr-318-Ile could be activated by Trp23-PTHrP while maintaining high potency for PTH(1–34). However, as indicated by the initial screen, TIP39 potency was compromised and reduced by almost 300-fold. Radioligand binding assays showed that the affinity of TIP39 had been reduced by >60-fold due to the Tyr-318-Ile mutation (Table 2).

### 3.3 Additional mutations at Tyr-318

**Fig. 3:**
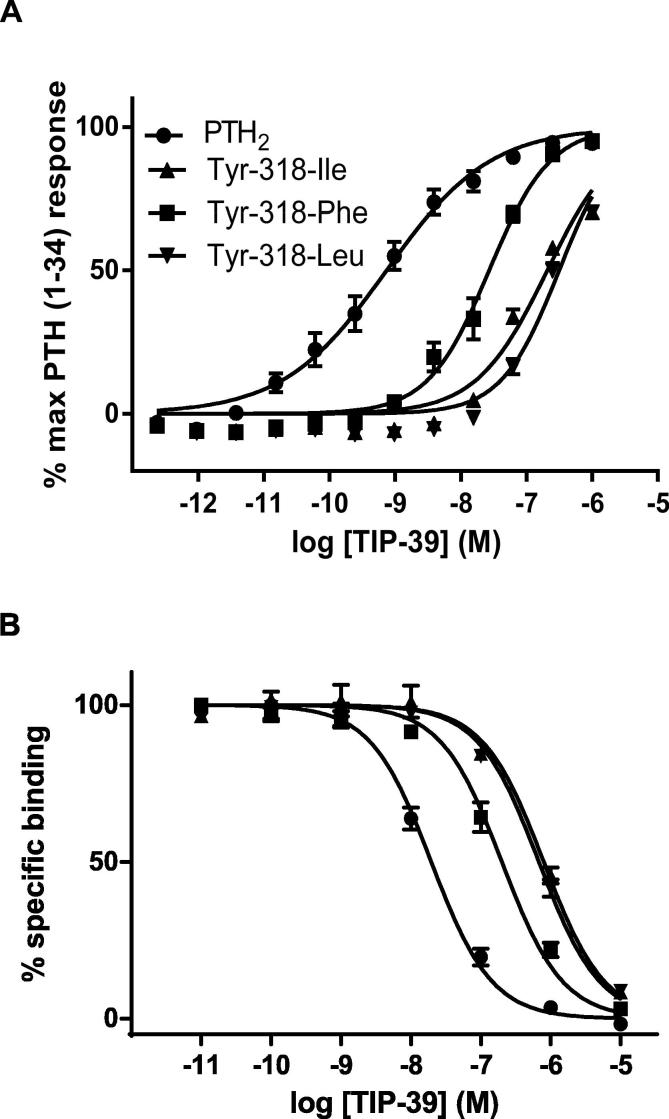
A. cAMP accumulation and B. radioligand competition binding assay, both using TIP39 at HEK-293 cells expressing PTH_2_, Tyr-318-Ile, Tyr-318-Leu or Tyr-318-Phe, as indicated in the key. Curves represent pooled data from three independent experiments where each point is the mean of the normalised values and inter-experimental standard error of the mean is displayed as error bars.

In order to explore the nature of the pharmacological effects caused by the mutation of Tyr-318 to Ile, the site was further mutated to both Phe and Leu. Radio-ligand binding and LANCE cAMP assays showed that the Tyr-318-Leu mutation resulted in a similar TIP39 profile as Tyr-318-Ile. However, as expected, the more subtle change from Tyr to Phe resulted in a less severe effect compared with the substitution to Ile upon TIP39 affinity (13-fold compared with 62-fold) and potency (35-fold compared with 282-fold). Nevertheless, the reduced affinity and potency of Tyr-318-Phe compared with PTH_2_ suggested that the hydroxyl group of Tyr-318 plays a role in endogenous ligand recognition (Table 2, Fig. 3).

### 3.4 N-terminal truncations of TIP39

**Fig. 4:**
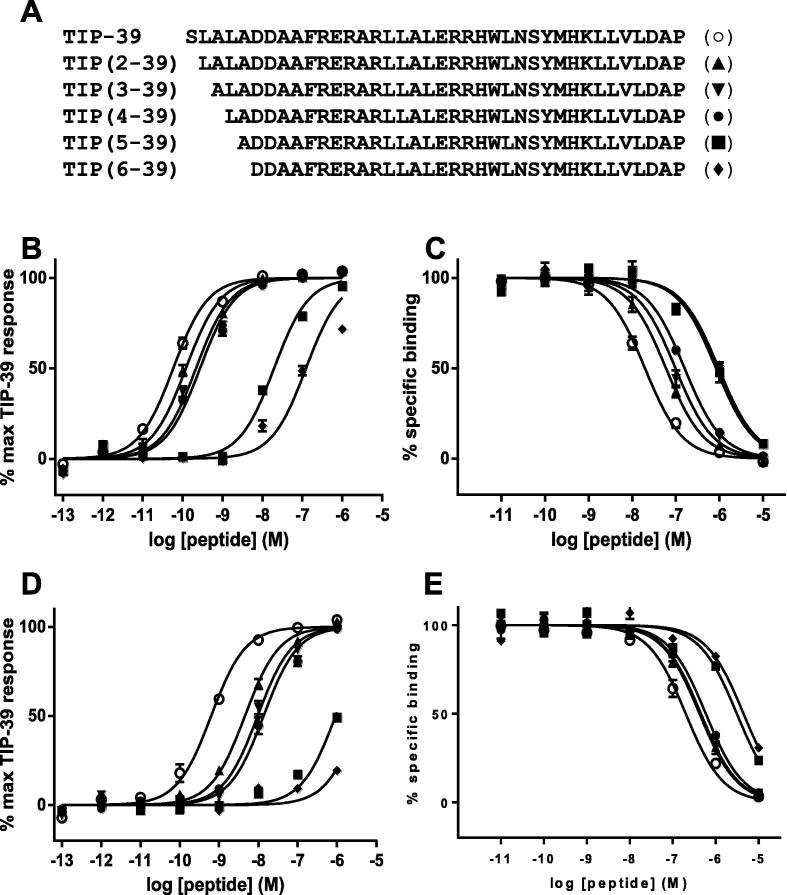
A Sequence alignment of TIP39 and the five N-terminally truncated analogues used in this study. B/D cAMP accumulation assays and C/E radioligand competition binding assays, both using HEK-293 cells expressing B/C PTH_2_ or D/E Tyr-318-Phe. The ligands used are indicated in the key in A. Curves represent pooled data from three independent experiments where each point is the mean of the normalised values and inter-experimental standard error of the mean is displayed as error bars.

In order to explore whether Tyr-318, and in particular its hydroxyl group, interacts with the N-terminal residues of TIP39, 5 N-terminal truncations of the peptide (Fig.4A) were pharmacologically analysed at PTH_2_, Tyr-318-Ile and Tyr-318-Phe (Table 3). Truncation of up to 3 residues at the N-terminus of TIP39 resulted in up to only a 5-fold reduction in potency but the removal of the 4th residue (Leu-4^∗^) resulted in a step-change reduction of almost 300-fold. Likewise, TIP39 missing the first 5 N-terminal residues had >2000-fold lower potency compared with full-length TIP39 (Fig.4B). The reduction in potency observed for peptides lacking residues 4 and 5, is only partly correlated with a reduction in affinity, with a much smaller step change (68-fold for affinity compared with 2138-fold for potency; Table 3; Fig.4B and C). The truncated TIP39 peptides displayed a similar profile at Tyr-318-Phe and Tyr-318-Ile, albeit with right-shifted curves, demonstrating that the mutations had affected all the peptides to a similar degree and that peptides lacking residue 4, or residues 4 & 5, have severely impaired potency at both the wild type and mutant receptors (Table 3, Fig. 4).

### 3.5 Molecular modelling and molecular dynamics simulations

**Fig. 5:**
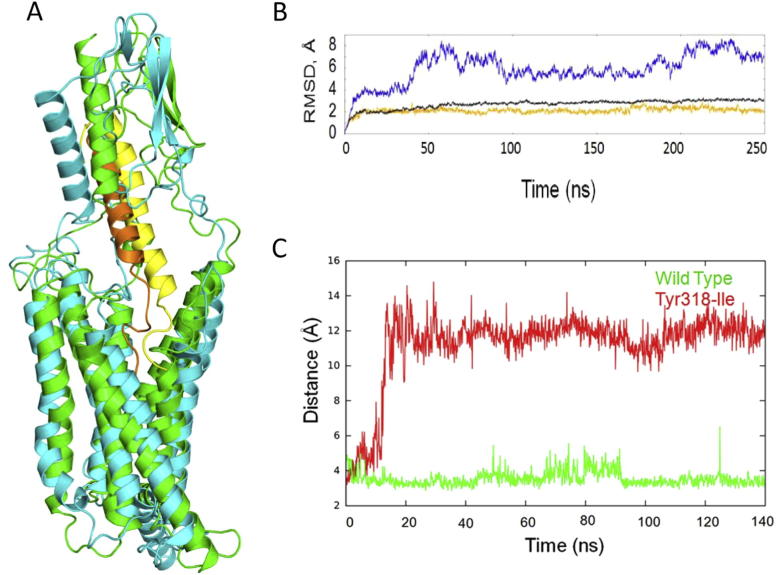
A. An overlay of the starting model of PTH_2_ bound to TIP (5–39) (green and orange), with the model following molecular dynamics simulations (cyan and yellow). B. Time dependent root mean square deviation (RMSD) of the backbone atoms for the full model (blue), the TM helices (black) and the N domain (orange) following molecular dynamics simulations (cyan and yellow in A) in relation to the starting structure (green and orange in A). C. Distance over time during molecular dynamics simulations between residue 318 (hydroxyl oxygen atom for Tyr or CD1 atom for Ile) and Asp-7 (CD atom) of TIP39 in the wild type (green) and mutant Tyr318-Ile (red) receptors. In Tyr318-Ile, Ile-318 does not interact with Asp-7 whereas in wild type Tyr318 and Asp-7 approached to form a stable hydrogen bond. (For interpretation of the references to colour in this figure legend, the reader is referred to the web version of this article.)

**Fig. 6:**
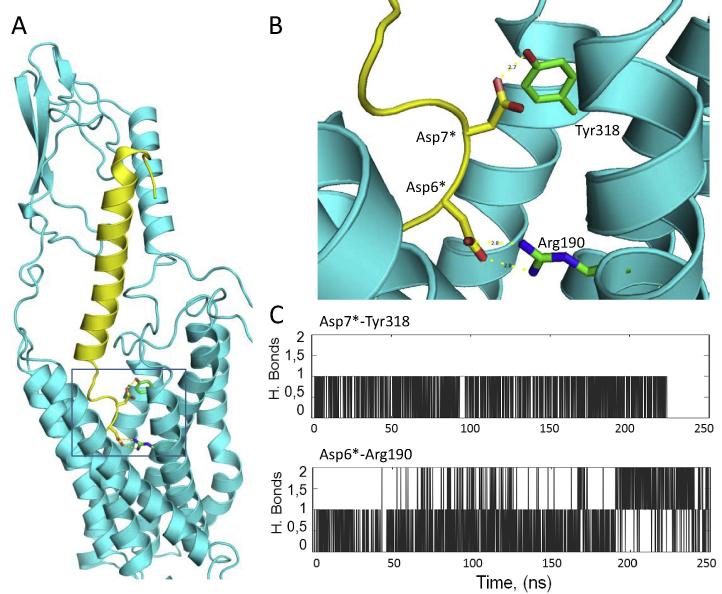
A. The model from molecular dynamics simulations of PTH_2_ bound to TIP (5–39), shown in ribbon form with PTH_2_ (cyan) docked with TIP-39 (yellow). The boxed area shows the region detailed further in B., showing interactions (dashed line with distances in Å) of Asp-6^∗^ and Asp-7^∗^ with the receptor. C. Time-dependent number of hydrogen bonds formed during molecular dynamics simulations of PTH_2_ bound to TIP (5–39) between the indicated residues. Cut off values for hydrogen bonds were donor acceptor distance <3.0 Å, and 20° for angle. (For interpretation of the references to colour in this figure legend, the reader is referred to the web version of this article.)

The initial starting model for the TIP39-PTH_2_ complex resembled that of the GLP-1R template used [16] in terms of the relative positioning of the N and J domains and on the placement of the helical region of the peptide ligand. However, while the N-terminal region of the ligand in the GLP-1R template was modelled on the β-coil region of the related ligand PACAP21 (pdb code 1GEA), the absence of sequence conservation in TIP39 and its longer length (3 residues longer than GLP-1) resulted in the need to build this region *ab initio,* with the guidance of two constraints. Firstly, the Glu-9^∗^ to Arg-190 interaction [16], [19] between GLP-1 and GLP-1R was emulated in the TIP-39-PTH_2_ model through an interaction between Asp-6^∗^-Arg-190 and, indeed, this interaction was maintained in the dynamics simulation (Fig.6C), in the same way as the analogous interaction was also stable in simulations of GLP-1R with GLP-1 [19]. Secondly, Ala-3^∗^ in TIP39 (equivalent to the first residue of PTH) was placed close to PTH_2_ residues 376, 379 and 380, since the extreme N-terminus of PTH has been cross-linked to these sites in PTH_1_ [10]. However, the ambiguity and sporadic nature of the initial simulations of full-length PTH_2_ bound to full-length TIP-39 (data not shown) led us to believe that the subjective modelling of the peptide’s extreme N-terminus was problematic. To address this, we prepared further simulations of PTH_2_ with a truncated N-terminus, TIP (5–39), which showed a stable pattern of hydrogen bonding of Tyr-318 with residue Asp-7^∗^ of TIP39 (Fig. 5, Fig. 6).

**Fig. 7:**
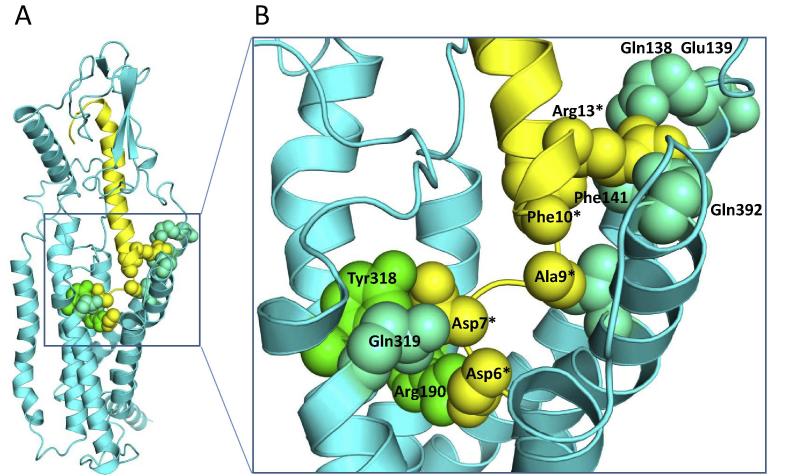
A. The model from molecular dynamics simulations of PTH_2_ bound to TIP (5–39), shown in ribbon form with PTH_2_ docked with TIP-39, and key interacting residues shown in space-fill. The boxed area shows the region detailed further in B. showing predicted interactions between PTH_2_ (cyan/green) and TIP-39 (yellow) which were see with high frequency during the simulation. (For interpretation of the references to colour in this figure legend, the reader is referred to the web version of this article.)

In addition to the Asp-6^∗^-Arg190 (2.60 × 60, [29]) interaction, the molecular dynamics predicted a number of other interactions in the TM domain binding pocket that were observed with significant frequency during the simulations (Fig. 7). For example, the hydrogen bond between Asp-7^∗^ and Tyr-318 (5.39 × 39) is supplemented by an additional interaction between the Asp-7^∗^ and Gln-319 (5.40 × 40). Arg13^∗^ can interact with either with Gln-138 (1.33 × 33) or Glu-139 (1.34 × 34) at the top of TM1, and also with Glu-392 (7.49 × 49) at the top of TM7. In addition, hydrophobic interactions were observed between Ala-9^∗^ and Leu-399 (7.43 × 42) and between Phe-10^∗^ and Phe-141 (1.36 × 36).

## 4 Discussion and conclusions

Both human PTH_1_ and PTH_2_ receptors are activated by PTH, suggesting that they share similar ligand binding sites and are activated by equivalent mechanisms. However, TIP39, the potent PTH_2_ agonist and endogenous PTH_2_ ligand, does not appreciably activate PTH_1_, despite binding to it with moderate affinity [21]. Chimeric PTH_1_/PTH_2_ receptors, in which the N and J domains were exchanged, have been used to demonstrate that it is the J domain which is responsible for interacting with the N-terminal region of TIP39 and for providing the observed selective binding [13], [14]. Hence, we have substituted selected PTH_2_ residues with those found in PTH_1_ and have identified Tyr-318 as the key residue position responsible for the observed reduction in reduced TIP39 activity. The results of the modelling and molecular dynamic simulations suggest that the N-terminus of the peptide binds within the helical bundle of the J domain, and that Asp-7^∗^ is the most likely interaction partner of Tyr-318 since its hydroxyl group can form an hydrogen bond with the carboxylate group of Asp-7^∗^, which persists during the MD simulations on the truncated peptide, TIP39(5–39), despite the dramatically reduced affinity of this peptide.

Residues predicted to be close to the extracellular ends of the TM regions of PTH_2_, and which were not conserved between PTH_1_ and PTH_2_, were targeted for site-directed mutagenesis. The start of this study preceded the solution of the first crystal structures of the J domain [5], [6], [7], and hence the TM boundaries were predictions but nevertheless largely match those revealed by the crystal structures (Fig. 1; TM boundaries shown are based on [6]). The 23 mutant receptors generated were screened using a cAMP assay in order to identify any mutations which had reduced TIP39 activity. As expected, given that both human PTH_1_ and PTH_2_ are activated by PTH, all the mutant receptors maintained full activity when exposed to 1 μM PTH. In hindsight, given the subsequent publication of the crystal structures, many of the residues targeted were facing away from the centre of the helical bundle and hence would be expected not to contribute directly to peptide binding. However, of the 6 internally-facing residues targeted for mutagenesis, only the Tyr-318-Ile mutation displayed reduced activity to 10 nM TIP39 and was hence examined in more detail.

Tyr-318-Ile in PTH_2_ has been identified previously as one of two sites responsible for enabling the receptor to select against PTHrP, probably through interacting with residue 5 of PTH and PTHrP [15]. Using Trp23-PTHrP (the Phe-23^∗^ to Trp modification enables higher affinity binding to PTH_2_ [13], [15]), the ability of the Tyr-318-Ile mutation to enable Trp23-PTHrP activation can be clearly observed using full concentration response curves (Fig. 2). However, we show for the first time here that the same mutation results in a substantial reduction in TIP39 potency (>280-fold) and affinity (>60-fold), yielding a receptor with substantially altered pharmacology from wild type PTH_2_ (compare Fig.2A and B).

In order to explore the mechanism underlying this altered pharmacological profile, we examined two further substitutions of Tyr-318, mutated to Phe and Leu, using radioligand binding assays and functional cAMP assays. Both Tyr-318-Leu and Tyr-318-Phe displayed high potency for PTH(1–34) and also rescued potency for Trp23-PTHrP, albeit both at a lower potency compared with Tyr-318-Leu, which has the substitution to the native PTH_1_ residue (Table 2). The Tyr-318-Leu receptor displayed similarly impaired TIP39 pharmacology as Tyr-318-Ile, with a TIP39 potency reduction of 437-fold and affinity reduction of 44-fold. The more subtle substitution of Tyr-318 with Phe, removing a single hydroxyl group, nevertheless resulted in a mutant receptor with 35-fold reduced potency and 13-fold reduced affinity for TIP39 compared with wild type PTH_2_. Hence we conclude that Tyr-318 takes part in an interaction that optimises the binding and activation pocket for TIP39 and that its hydroxyl group forms part of this interaction.

Given that the truncation of the first 6 residues of TIP39, to yield TIP (7–39), resulted in a peptide with no observable efficacy at PTH_2_ [21], we explored the possibility that the interaction formed by Tyr-318 may be via these first 6 residues. Indeed, the removal of 4 or 5 residues from TIP39 resulted in a peptide with a reduced affinity that approximates (about 60-fold) to that observed when the hydroxyl group of Tyr-318 is removed (Table 2, Table 3). We hypothesised that a direct interaction between PTH_2_ and a particular N-terminal residue on the ligand would be revealed if the removal of that residue led to no detectable change in affinity/potency when comparing wild type PTH_2_ and a Tyr-318 mutant: – the logic being that if the interacting residue was already absent from the ligand (due to truncation), then the removal of the Tyr-318 interaction site would result in no additional change in affinity/potency. However, the Tyr-318-Ile and Tyr-318-Phe mutations affected all the truncated TIP39 analogues used, including TIP (6–39), suggesting that Tyr-318 interacts with a residue within the 6–39 region. The absence of potency for TIP39 peptide analogues truncated beyond this point [21] makes it difficult to explore this possibility experimentally, and hence we have used a computational approach to explore the hypothesis that Tyr-318 may interact with Asp-7^∗^, the topologically equivalent site to reside 5^∗^ of PTH and PTHrP that has previously been shown to be functionally linked to Tyr-318 [15]. There had been an earlier attempt [28] to model TIP39 binding to PTH_2_ which suggested that Asp-7^∗^ interacts with His-396 on TM7. However, this study pre-dated the solution of the structure of any Family B J domain and the new information suggests that His-396 is positioned at the TM1-TM7 interface and quite distant from Asp-7^∗^. Our modelling has enabled the predictions of a dual interaction between Asp-6^∗^ and Arg-190 and between Asp-7^∗^ and Tyr-318 which, coupled with the cross-linking data [8], [9], [10], makes the PTH_1_/PTH_2_ system particularly well characterized for class B receptors. Truncation of Asp-6^∗^ led to a complete loss of activity [21] and our model suggests it plays a key role through binding to Arg190 – indeed, this interaction may be quite general amongst class B GPCR ligands as several have a residue similar to Asp-6^∗^ at this position [30].

In summary, we have analysed 23 sites within the PTH_2_ receptor and identified Tyr-318 as playing an important role in the interaction of the endogenous TIP39 agonist with its receptor, given that its substitution with Ile or Leu results in a 282–437-fold reduction in potency and a 44–62-fold reduction in affinity. The hydroxyl group of Tyr-318 is involved in this interaction since its removal resulted in a 35-fold reduced potency and 13-fold reduced affinity for TIP39. To our knowledge, this is the first reported PTH_2_ receptor residue shown to interact with its endogenous agonist TIP39 and its identification, alongside our model based upon the latest crystallographic data for Family B GPCRs, enabled us to suggest a binding model for the N-terminus of TIP39 within the TM domain of PTH_2_. The interaction of Tyr-318 is likely to be with the N-terminal region of TIP39, although not with residues 15. Given the outcome of the molecular modelling and molecular dynamics study, coupled with alignment of Asp-7^∗^ in TIP39 with His-5^∗^ in PTHrP and Ile-5^∗^ in PTH, we propose that the most likely candidate to interact with Tyr-318 is Asp-7^∗^. The simulations also identified Asp-6^∗^ as a likely binding partner of Arg-190, as also observed in GLP-1R [16] and in the VPAC receptor [31], [32].

## Author contributions

REW – carried out molecular biology and pharmacological assays, contributed to writing the paper. JCM & CAR – advised on model construction, planned and carried out the molecular dynamics simulations and revised the manuscript critically for important intellectual content; MJW – planned and supervised the pharmacological screen of the mutant receptors, and revised the manuscript critically for important intellectual content; DD – designed and made the TIP39-PTH2 starting model, planned and supervised the project, wrote the paper.

## Figures and Tables

**Table 1:** cAMP assays of wild type PTH_1_ and PTH_2_, and 23 mutated PTH_2_ receptors substituted at the sites indicated in Fig. 1 (single letter amino acid codes are shown here with GPCRDB numbering in column 2 [29]). Values are the% of the mean maximal PTH(1–34) response using either 1 μM PTH(1–34) *left*, or 10 nM TIP39 *right*. One residue (Tyr-318-Ile) displays reduced TIP39 activity. ^**^Significantly different to PTH_2_ (*P* ⩽ 0.002).

Receptor	GPCRDB numbering	% maximum PTH(1–34) response	
		1 μM PTH(1–34)	10 nM TIP-39
**PTH_1_**		**99** **±** **2**	**1** **±** **2**
**PTH_2_**		**102** **±** **1**	**98** **±** **2**
PTH2R (V146 M)	1.41 × 41	102 ± 1	103 ± 1
PTH2R (M147I)	1.42 × 42	103 ± 2	100 ± 2
PTH2R (I154V)	1.49 × 49	104 ± 3	103 ± 5
PTH2R (V185L)	2.55 × 55	105 ± 2	104 ± 4
PTH2R (T192V)	2.62 × 62	102 ± 2	99 ± 1
PTH2R (I238V)	3.31 × 31	104 ± 2	97 ± 2
PTH2R (V241T)	3.34 × 34	100 ± 1	97 ± 2
PTH2R (M242F)	3.35 × 35	98 ± 2	98 ± 2
PTH2R (I244L)	3.37 × 37	100 ± 3	106 ± 4
PTH2R (A293V)	4.59 × 59	99 ± 2	97 ± 1
PTH2R (A295V)	4.61 × 61	99 ± 2	98 ± 1
PTH2R (V296S)	4.62 × 62	101 ± 2	101 ± 3
PTH2R (A297V)	4.63 × 63	100 ± 2	99 ± 1
PTH2R (Y318I)	5.39 × 39	99 ± 1	**51** **±** **6**^∗∗^
PTH2R (A320V)	5.41 × 41	100 ± 1	97 ± 2
PTH2R (A325S)	5.46 × 46	103 ± 2	100 ± 2
PTH2R (G327V)	5.48 × 48	100 ± 2	99 ± 2
PTH2R (V380 M)	6.57 × 57	102 ± 1	102 ± 1
PTH2R (C381A)	6.58 × 58	100 ± 2	101 ± 2
PTH2R (L382T)	ECL3	102 ± 2	103 ± 2
PTH2R (C397Y)	7.41 × 40	100 ± 2	98 ± 1
PTH2R (L399M)	7.43 × 42	101 ± 1	98 ± 2
PTH2R (F400L)	7.44 × 43	104 ± 2	105 ± 3

**Table 2:** Pharmacological data for various peptide ligands at wild type PTH_2_ and three mutated PTH_2_ receptors, as indicated. Values represent mean pEC_50_ and pIC_50_ values ± S.E.M. for three independent experiments, with the corresponding EC_50_ or IC_50_ values (nM) shown below in brackets. B_max_ were derived from three independent homologous radioligand competition binding assays using the radioligand ^125^I-rPTH(1–34). Significantly different from PTH_2_ with the TIP-39: ^*^P ⩽ 0.02; ^**^P ⩽ 0.002).

	pEC_50_ (EC_50_/nM)			pIC_50_ (IC_50_/nM)	B_max_ /amol cell^−1^
	PTH(1–34)	Trp23-PTHrP	TIP-39	TIP-39	
PTH_2_	8.39 ± 0.28 (4.1)	ND	9.14 ± 0.35 (0.7)	7.81 ± 0.02 (15.5)	13.3 ± 2.4
Tyr-318-Phe	8.17 ± 0.30 (6.8)	5.84 ± 0.15 (1445.4)	7.60 ± 0.22^∗^ (25.1)	6.70 ± 0.16^∗∗^ (199.5)	10.7 ± 2.4
Tyr-318-Leu	8.66 ± 0.22 (2.2)	6.09 ± 0.20 (812.8)	6.50 ± 0.09^∗∗^ (316.2)	6.17 ± 0.08^∗∗^ (676.1)	12.3 ± 2.2
Tyr-318-Ile	9.45 ± 0.08 (0.4)	7.39 ± 0.08 (40.7)	6.69 ± 0.03^∗∗^ (204.2)	6.02 ± 0.03^∗∗^ (955.0)	21.3 ± 7.7

**Table 3:** Pharmacological data for TIP39 and N-terminally truncated analogues (see Fig. 4A) at wild type PTH_2_ and two mutated PTH_2_ receptors, as indicated. Values represent mean pEC_50_ and pIC_50_ values ± S.E.M. for three independent experiments, with the corresponding EC_50_ or IC_50_ values (nM) shown below in brackets. Significantly different from TIP39 at the same receptor: ^∗^ P ⩽ 0.02; ^∗∗^ P ⩽ 0.002).

	pEC_50_ (EC_50_/nM)		
	PTH_2_	Tyr-318-Phe	Tyr-318-Ile
TIP-39	10.24 ± 0.07	9.18 ± 0.04	8.18 ± 0.09
	(5.75 × 10^−2^)	(0.66)	(6.61)
TIP (2–39)	9.92 ± 0.10	8.32 ± 0.11^∗∗^	7.38 ± 0.17^∗^
	(0.12)	(4.79)	(41.69)
TIP (3–39)	9.66 ± 0.13^∗^	8.02 ± 0.11^∗∗^	7.30 ± 0.17^∗^
	(0.22)	(9.55)	(50.12)
TIP (4–39)	9.54 ± 0.14^∗^	7.88 ± 0.20^∗^	7.05 ± 0.14^∗^
	(0.29)	(13.18)	(89.13)
TIP (5–39)	7.77 ± 0.06^∗∗^	6.04 ± 0.18^∗∗^	<6
	(16.98)	(912.01)	(>1000)
TIP (6–39)	6.91 ± 0.16^∗∗^	<6	ND
	(123.03)	(>1000)	

	pIC_50_ (IC_50_/nM)		
	PTH_2_	Tyr-318-Phe	Tyr-318-Ile

TIP-39	7.81 ± 0.02	6.70 ± 0.16	6.02 ± 0.03
	(15.49)	(199.53)	(954.99)
TIP (2–39)	7.23 ± 0.15^∗^	6.38 ± 0.10	5.83 ± 0.12
	(58.88)	(416.87)	(1479.11)
TIP (3–39)	6.98 ± 0.10^∗∗^	6.32 ± 0.09	5.90 ± 0.11
	(104.71)	(478.63)	(1258.93)
TIP (4–39)	6.80 ± 0.04^∗∗^	6.22 ± 0.11	5.83 ± 0.13
	(158.49)	(602.56)	(1479.11)
TIP (5–39)	6.06 ± 0.08^∗∗^	5.49 ± 0.10^∗^	5.14 ± 0.08^∗∗^
	(870.96)	(3235.93)	(7244.36)
TIP (6–39)	5.98 ± 0.07^∗∗^	5.37 ± 0.09^∗∗^	5.06 ± 0.04^∗∗^
	(1047.13)	(4265.78)	(8709.63)
